# The association of host genes with specific sexually transmitted infections

**DOI:** 10.3389/frph.2023.1124074

**Published:** 2023-10-23

**Authors:** Qhama Bovungana, Thilona Arumugam, Veron Ramsuran

**Affiliations:** ^1^ School of Laboratory Medicine and Medical Sciences, College of Health Science, University of KwaZulu-Natal, Durban, South Africa; ^2^ Centre for the AIDS Programme of Research in South Africa (CAPRISA), University of KwaZulu-Natal, Durban, South Africa

**Keywords:** sexually transmitted infections, toll-Like receptors, host genetics, host factors, pathogen recognition receptor

## Abstract

Sexually transmitted infections (STIs) are hazardous to human health worldwide. STIs have a direct influence on sexual and reproductive health and can increase the chances of HIV. Globally, more than 1 million STIs are acquired every day and the majority are asymptomatic. Approximately, 374 million cases of STIs have been reported annually. The most prevalent STIs include chlamydia, gonorrhoea, syphilis, and trichomoniasis. These STIs are caused by *Chlamydia trachomatis*, *Neisseria gonorrhoeae*, *Treponema pallidum* and Trichomonas vaginalis. The major factor that contributes to the susceptibility and prognosis of infectious diseases is genetic variation. Host genes play a huge role in STIs and immune response. The production of host factors is stimulated by a variety of bacteria, viruses and parasites and the host factors can play a role in increasing host vulnerability to infection and pathogen persistence. Genetic variation or polymorphisms within certain host genes can influence the course of pathogen infection and disease progression. Polymorphisms can contribute to changes in gene expression and or changes in the protein structure. which may either contribute to/or protect against infection. This review discusses the role of host genes in influencing the susceptibility of the most prevalent STIs caused by *Chlamydia trachomatis*, Trichomonas vaginalis, *Treponema pallidum* and *Neisseria gonorrhoeae*. We evaluate polymorphisms associated pathogen recognition signalling pathway of these diseases. These polymorphisms may be used as biomarkers to infer risk to specific STIs.

## Introduction

Sexually transmitted infections (STIs) are detrimental to sexual and reproductive health worldwide. Globally, they are the most prevalent acute infections. There are more than 30 infections that can be spread during sex (1). According to the World Health Organization (2), four major STIs that contribute highly to the annual statistics: chlamydia (129 million), gonorrhoea (82 million), syphilis (7.1 million), and trichomoniasis (156 million) cases (3). Genital infections primarily affect the lower urogenital tract which inflames the vagina, cervix, urethra, or penis. However, most genital infections are asymptomatic, self-limiting conditions that frequently go undiagnosed. Even so, the prolonged term of untreated STIs may lead to diseases such as pelvic inflammatory disease (PID), tubal infertility (TF) and onward transmission (4).

During infection, the first line of defence is the innate immune response which can differentiate between structural components and microbial pathogens that are present only in these microorganisms and are absent in the normal host cells, this brings about an immune response. In the innate immune response, studies have shown that mucosal host gene production takes place after the acquisition of an STI and is a key component of the subsequent immune response (5). Then, the adaptive immune response is activated by the innate responses, and they combine to destroy the infections. The adaptive immune responses, in contrast to innate immune responses, are very particular to the pathogen that triggered them. They may also offer a prolonged defence. For instance, if re-infection happens in some STIs a long time after the first infection, as the adaptive immune response that follows the primary infection is necessary for immunological surveillance and serves as the first line of defence against secondary infection (5).

**Figure 1:**
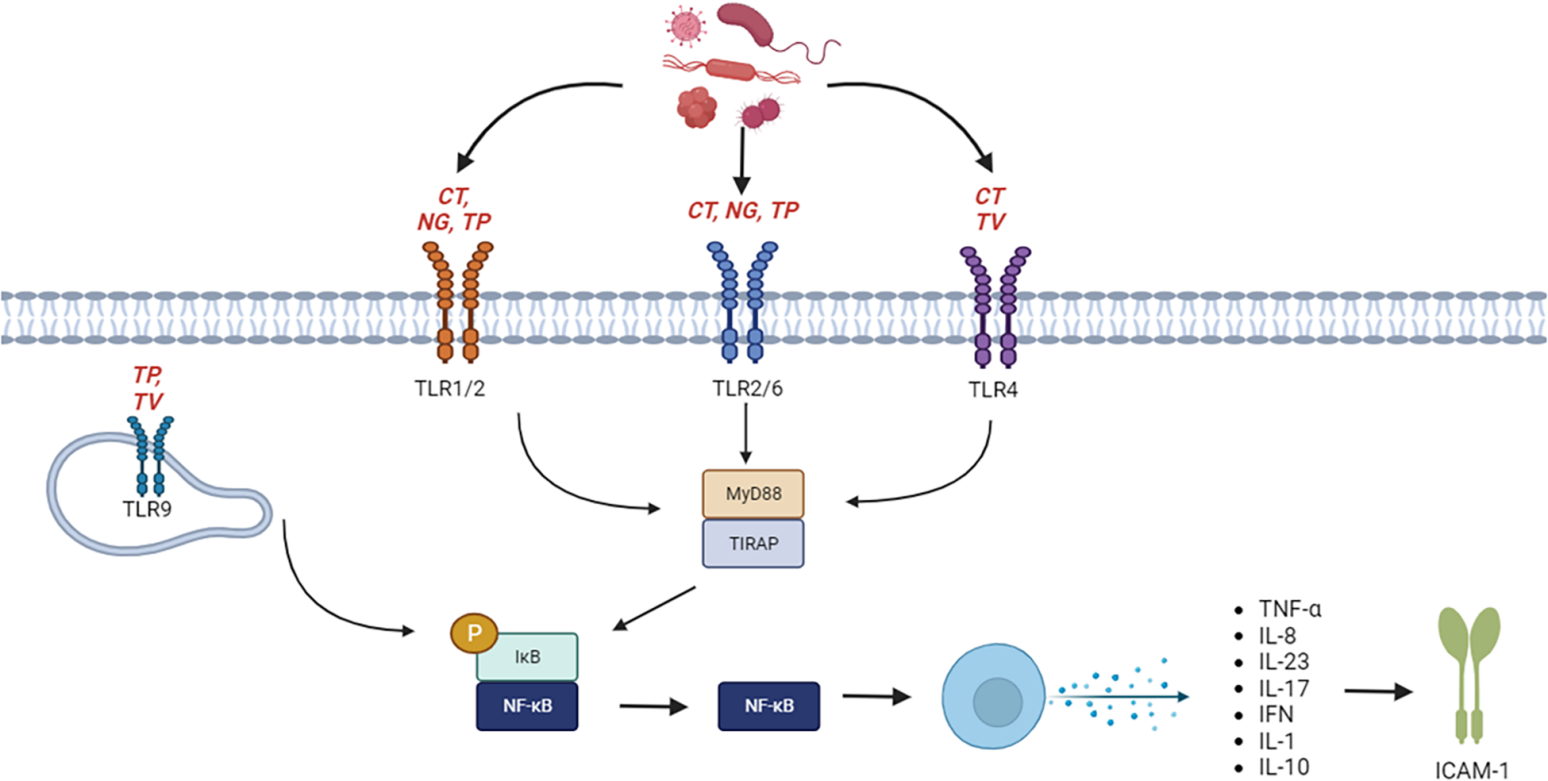
Schematic overview of STIs stimulating the innate immune response and induction of pro-inflammatory responses.

The activation of the immune response initiates the host gene response. These genes include Toll-like receptors (TLRs), chemokine ligands (CCLs), interleukins (ILs), nodulation-like receptors (NLRs), which will be discussed in this review. One of the primary innate immune responses is the family of TLRs, they then lead to a cascade of events to initiate the regulation of genes such as Intercellular adhesion molecule-1 (ICAM-1) (6). TLRs are the main innate immune response components that have been linked to numerous STIs. TLR1, TLR2, TLR4, TLR5, TLR6 and TLR10 are cell surface receptors, whereas TLR3, TLR7, TLR8, and TLR9 are receptors of an endosomal compartment (6). TLRs respond to the pathogen designated for it. For instance, TLR1/TLR2 and TLR2/6 complex is stimulated by heat shock protein 60 (HSP60) and lipo-oligosaccharide of *Neisseria gonorrhoeae* (NG), major outer membrane proteins of Chlamydia trachomatis (CT) and lipoprotein of Treponema pallidum (TP); *TLR4* is stimulated by lipid A which forms part of the lipopolysaccharide moiety that is located on the outer membrane of gram-negative bacteria such as CT a major outer membrane protein of CT and HSP60/70, fibronectin and CpG of Trichomoniasis vaginalis (TV). TLR9 is stimulated by CpG d TV and dsRNA of TP (7). Upon stimulation signalling is mediated either via myeloid differentiation primary response 88 (MyD88) pathway or (TIR-domain-containing adapter-inducing interferon-*β* (TRIF) pathway. Both pathways lead to the activation of NF-κB and the release of pro-inflammatory cytokines. High levels of pro-inflammatory cytokines stimulate the expression of ICAM-1 which facilitates a variety of immune responses (Figure 1).

Genes associated with pathogen recognition signalling can be used as biomarkers. For instance, the levels of *ICAM-1* mRNA were evaluated as potential biomarkers for the inflammatory response in vaginal cells (8). Genetic variation within immune genes may alter protein structure/activity and change gene expression, thus altering immune response. For instance, genetic variations such as single nucleotide polymorphisms (SNPs) have a significant impact on innate immune responses to pathogenic challenges and disease outcomes; as a result, people have varying levels of susceptibility to infections, with some being predisposed to certain infections and others being protected. Although pathogen genetic diversity has a significant impact on disease outcome, host genetics also have a role in the pathogen-host interaction (9). Recent developments in the field of STIs and host genes globally have led to a renewed interest in host genes associated with the most prevalent STIs caused by CT, TP, TV and NG. This review discusses SNPs associated with pathogen recognition in the above-mentioned genes. We summarize host genetic studies conducted on sexually transmitted infections and some of their complications (Table 1).

**Table 1:** Host SNPs associated with STI recognition immune signalling pathway.

Pathogen	Gene	SNP	Disease	Population	Frequency of alleles (associated study)	Reference
* Chlamydia trachomatis*	*TLR1*	rs5743618 G>T	CT infection	African American Women (*n* = 205)	Wildtype = 0.23 Mutant = 0.77	(10)
	*TLR4*	rs1927911 T>C	PID	African American Women (*n* = 205)	Wildtype = 0.49 Mutant = 0.51	(10)
	*TLR2*	−16934T/+2477G –1237C	CT infection	Caucasian Women (CT^+^ *n* = 322; CT^−^ *n* = 409)	Wildtype = 0.50 Mutant = 0.499/Wildtype = 0.95 Mutant = 0.0497	(11)
	*TLR9*	−1237T/+2848A	CT infection	Caucasian Women (CT^+^*n* = 322; CT^−^ *n* = 409)	Wildtype = 0.47 Mutant = 0.53	(11)
	*NOD1*	rs6958571	CT infection	Caucasian Women (CT^+^ *n* = 23; CT^−^ *n* = 409)	Wildtype = 0.85 Mutant = 0.15	(12)
	*NOD1*	+32656T>G	CT infection	Caucaian Women (CT^+^ *n* = 724; CT^−^ *n* = 282)	Wildtype = 0.81 Mutant = 0.19	(13)
	*IL10*	−1082 A>G	Tubal factor infertility	Caucasian Women (CT^+^ *n* = 724; CT^−^ *n* = 282)	Wildtype = 0.54 Mutant = 0.46	(13)
* Neisseria gonorrhoeae and or Chlamydia trachomatis*	*TLR1*	rs5743618 C>A	PID	African Women (CT and/or NG =205); Caucasian Women (CT and/or NG = 51)	African American GG + GT =0.271 TT = 0.729 Caucasian GG + GT =0.786 TT = 0.214	(14)
	* *	rs4833095 T>C			African American AA + AG =0.398 GG = 0.602 Caucasian AA + AG =0.851 GG = 0.149	
	*TLR2*	rs3804099 T>C			African American TT =0.16 CT = 0.393 CC = 0.447 Caucasian TT = 0.292 CT = 0.500 CC = 0.208	
	*TLR4*	rs4986790 A>G			African American TT = 0.841 CT = 0.159 Caucasian TT = 0.900 CT = 0.500 CC = 0.208	
	*TLR6*	Rs5743810 A>G			African American CC =0.830 TT + CT = 0.170 Caucasian CC =0.404 TT + CT = 0.596	
	*TIRAP*	Rs8177374 C>T			African American CT =0.051 CC = 0.949 Caucasian CT = 0.272 TT + CT = 0.723	
* Treponema palladium*	*IL17*	rs2275913A/rs3819024G	Neurosyphilis	Asian (East) Women (TP^+^ *n* = 118; TP− *n* = 216)	Wildtype = 0.53 Mutant = 0.47 Wildtype = 0.52 Mutant = 0.48	(15)
	*TLR2*	rs5743708 (2258G→A)	Syphilis	European Men and Women (TP + *n* = 137; TP − *n* = 221)	Wildtype = 0.99 Mutant = 0.01	(16)
* Trichomonas vaginalis*	*TLR4*	rs11536889 CC	Cervicitis	Asian (South) Women (TV^+^ *n* = 130; TV^−^ *n* = 150)	Wildtype = 0.775 Mutant = 0.225	(17)
	*TLR4*	rs4986790 A>G rs4986791 C>T	Nill	Middle Eastern Women (TV^+^ *n* = 80; TV^−^ *n* = 52)	Wildtype = 0.92 Mutant = 0.8 Wildtype = 0.943 Mutant = 0.575	(18)
	*TLR9*	rs187084 TC	Cervicitis	Asian (South) women (TV^+^ *n* = 130; TV^−^ *n* = 150)	Wildtype = 0.885 Mutant = 0.115	(17)

## Host factors associated with sexually transmitted infections

### Host genetic factors associated with *Chlamydia trachomatis* infections

The health of human sexual and reproductive systems is extremely affected by CT infections. They are regarded as the major global contributors to bacterial STIs (1). CT is a gram-negative, obligatory, and immobile intracellular bacterium that causes Chlamydial infection. In 2020, CT prevalence worldwide was found to be 4.2% for women and 2.7% for men (19). Although the infection may be asymptomatic, it can nonetheless manifest as vaginal discharge, irregular vaginal bleeding, lower pelvic pain, frequent urination, or dysuria (20).

A considerable amount of literature has been published on CT infections and data from several studies have identified the effect of host genes and chlamydial infections (9, 21, 22). Variation within host genes is associated with either increased susceptibility or protection against infections. When CT enters the host, a variety of host factors such as receptors are triggered for immune response. Such host factors include the TLR family. Previously, TLRs emerged as a vital component of the innate immune system that recognizes microbial infection and initiates host defence responses against microbes (23). During pathogen invasion, TLRs are key mediators of inflammatory pathways in the gut, mediating immune responses to a variety of ligands originating from pathogens and linking innate and adaptive immunity (24). Several bacterial STIs interact with extracellular TLRs to trigger innate inflammatory reactions. The interaction of these receptors may lead to the induction of apoptosis (25).

Studies have associated certain SNPs in TLRs with CT acquisition and progression (26, 27). For instance, in African American women, rs5743618TT (*TLR1*) was associated with Chlamydial infection which has led to PID complications (28). The polymorphisms in these receptors have been associated with multiple CT infections. As shown in Table 1, a study performed in African American women, (*TLR1*) rs5743618TT was associated with CT infection and women with PID who had rs1927911 CC (*TLR4*) and had high chances of contracting the CT infection (10, 14). The rs5743618TT (*TLR1*) has a nucleotide base change from 1805 G>T and leads to the change in the amino acid on the cytoplasmic side of the transmembrane domain of the receptor, from serine (Ser) to isoleucine (Ile). The change in the amino acid may modify the protein, leading to the change in the protein expression hence resulting in susceptibility to CT infection.

Furthermore, TLR2 and TLR4 are usually associated with CT infections since they recognize LPS, outer membrane vesicles, porins, and other proteins, and NOD1 and NOD2 recognize other STI biochemical compounds such gamma glutamyl diaminopimelic acid and muramyl dipeptide, which also stimulates NF-KB-driven inflammation (29). Nonetheless, genetic polymorphisms may vary across different ethnic groups. In Caucasian women, (*TLR2*) haplotype I (−16934T/+2477G) showed protection against the development of chlamydial symptoms and tubal pathology after Chlamydia infection (11). Also, in the same cohort of Caucasian women, (*TLR9*) haplotype III (–1237C/+2848A) showed a substantial decrease in the start of symptoms after CT infection.

TLRs, sometimes require Myeloid differentiation primary response 88 (MyD88) for signalling. MyD88 is an essential intracellular protein adaptor molecule that induces nodulation-like receptors (NLRs) response. The TLRs then interact with many other proteins to trigger a cascade of immune responses. For instance, most TLRs except TLR3 require MyD88 pathway to bring about an immune response (30). The pathway induces the NOD1 and NOD2 response. Specialized NLRs called nucleotide-binding and oligomerization domains (NOD1 and NOD2) are involved in the identification of a subset of pathogenic microorganisms that can infiltrate and proliferate intracellularly. A subset of inflammatory genes is expressed when these molecules are active, activating intracellular signalling that in turn stimulates transcriptional responses (31). Literature shows that NOD2 detects both gram-positive and gram-negative bacteria and NOD1 detects a wide range of gram-negative bacteria (32). As a result, in a study performed on Dutch Caucasian women, +32656 GG rs6958571 (*NOD1*) was associated with CT infection (12).

IL-10 is a strong anti-inflammatory cytokine that is important for controlling the host immune system's response to infections (33). Various *IL-10* gene promoter polymorphisms have been linked to both disease severity and prevalence, and IL-10 has been found to affect both disease susceptibility and progression. A study on women from five cohorts of West-European ethnicity evaluated the association of polymorphisms within four genes involved in microorganisms' detection and initiation of inflammatory responses. The +32656T>G (*NOD1*) associated with symptomatic CT infection (Jukema et al., 2021) and −1082 A>G, a polymorphism on IL-10 increased the chance of late complications such as tubal factor infertility. Studies have indeed shown that the association of CT infections with host factors influences the susceptibility to infection and disease progression.

### Host genetic factors associated with *Neisseria gonorrhoeae* infections

Gonorrhoea is caused by a gram-negative diplococci bacterium which is known as *Neisseria gonorrhoeae* (NG). The bacterium uses glucose to invade mucus epithelial cells. Gonorrhoea is the second most common STI after CT infection (34). If untreated, gonorrhoea is one of the major causes of PID and infertility in women. Non-Caucasian women were shown to have a higher risk of contracting upper urogenital tract NG and/or CT infection. Ethnic disparities have been associated with PID-associated CT and NG. Polymorphisms within the TLR family and the TLR adaptor molecule, Toll/interleukin-1 receptor domain-containing adaptor protein (TIRAP) are a risk factor for this disparity. African American women had a higher frequency of rs5743618 TT (*TLR1*) and rs4833095 GG (*TLR1*) compared to Caucasian women. Similarly, rs3804099 CC (TLR2) and rs8177374 CC (TIRAP) were more frequent in African Americans than Caucasian women. In contrast, African American women were significantly less likely to carry a T allele for rs5743810 (TLR6). No significant differences were found for the rs4986790 (TLR4). African American women with T allele for rs5743810 (TLR6) had a decreased risk of upper urogenital tract NG and/or CT infection. A similar trend was seen in Caucasians, but this was not significant. African Americans who carried the rs5743618 TT (TLR1), rs4833095 GG (TLR1) and rs8177374 CC (TIRAP)had a higher risk of contracting upper urogenital tract infection, while Caucasian women with the rs3804099 CC (TLR2) had a higher risk compared to women who carried the CT or TT genotypes (10, 14).

The invasion of a pathogen in the epithelial cells leads to an overexpression of host genetic factors. There is little information published on the host factors associated with NG infection. In the few studies of NG, researchers have evaluated the expression of host factors and not much is known about the association of alleles with NG progression and acquisition. Hence, studies examined the expression of ICAM-1.

Invasion of NG in the vaginal cells increases ICAM-1 level (35). Despite that, not much research = has been published on NG infections that are associated with genetic variation within hosts. Also, in a study performed on ICAM-1, none of the alleles were shown to predispose an individual to NG infection but they investigated how epithelial cells expressed ICAM-1 during NG infection. Even though some vaginal infections are not sexually transmissible but are induced by a microflora imbalance, these infections may upregulate the prevalence of host genes involved, increasing the chances of STIs. For example, ICAM-1 has been associated with the vulvovaginal candidiasis infection related to diabetic patients which leads to *Candida* susceptibility (36). Moreover, underlying diseases may also lead to increased susceptibility to infections.

Furthermore, in a study where gonorrhoea was found in individuals, *IL-10* and *IL-12* concentrations were higher and *IL-2* concentrations were lower among the three endocervical cytokines tested. The author's interpretation overlooks much after gonorrhoea acquisition, patients with the *IL-2*T-G haplotype were the only ones who had a decrease in endocervical *IL-2*, indicating that a pathogen-specific and genetically mediated mechanism for distinct *IL-2* responses at the genital mucosa is stimulated (37). Very little is known about the role of host genetic factors in gonorrhoea infection and therefore, there is a need to investigate other host genetic factors.

### Host genetic factors associated with *Treponema pallidum* infections

Syphilis is caused primarily by *Treponema pallidum*, which is a multisystem infection. It is often transferred sexually, but it can also be transmitted from a parent to an offspring during pregnancy, resulting in congenital syphilis. Globally, there were an estimated 7 million new syphilis infections in 2020 (3). Every year, roughly six million new cases of syphilis are diagnosed in people aged 15–49 years old worldwide (38). Syphilis presents in various forms of infection, depending on the duration of infection known as primary, secondary, or tertiary. In primary infection, syphilis causes a single or tiny, painless sore in or around the mouth, genitalia, or anus. If the primary stage is untreated it progresses to the secondary stage may result in the tertiary stage which leads to neurosyphilis. Hence, not much investigation was done on syphilis and neurosyphilis. Only a few studies examined the association of SNPs with the acquisition and progression of the disease. SNPs in eight genes were examined in a study that involved 188 Han Chinese patients with syphilis. These genes included the vitamin D receptor (VDR), a member of the nuclear receptor family of transcription factors, and interleukins (ILs), which act as both pro-inflammatory cytokines and anti-inflammatory myokines. The study also examined chemokine ligands (CCLs) that bind to multiple chemokine receptors (CCRs). The genes which were examined were *IL-17A*, *IL-17F*, *IL-23R*, *VDR*, *CCL2*, *CCL5*, *CCR2*, and *CCR5*. The findings showed a significant correlation of *IL-17*A rs2275913 (AA vs. AG + GG) and rs3819024 (GG vs. AA + GA) for those with syphilis. The *IL-17*A rs2275913A/rs3819024G haplotype showed a risk effect in haplotype analysis, while *IL-17*A rs2275913G/rs3819024A showed a protective effect (15).

As mentioned previously, untreated syphilis spreads in the body, resulting in neurosyphilis. For instance, in 456 Caucasian syphilis patients, polymorphisms in the T>G +1805 (*TLR1*), G>A +2258 (*TLR2*), and C>T +745 (*TLR6*) were looked for. Laboratory-defined neurosyphilis is associated with a common *TLR1* polymorphism, while clinically and laboratory-defined neurosyphilis are both associated with common *TLR2* and *TLR6* polymorphisms (39). Most of the *TLR* SNPs that are intronic and one that is synonymous are associated with TP infection outcomes; like most SNP-association studies, these SNPs will need more research to see how they relate to functional traits that affect TP susceptibility. For instance, since mRNA splicing requires splice donor and acceptor sites, exon splicing enhancers, and splicing proteins, intronic SNPs may have an impact on gene regulation or splicing. Splice variants are created when the activity of mRNA splicing is modulated by sequence changes brought on by synonymous or non-synonymous SNPs (40). Also, the tagSNPs that are in linkage disequilibrium with a causative SNP may be the cause of associations between non-functional SNPs and TP infection.

In another study, as shown in Table 1, the role of rs5743708 (*TLR2*) 2258G → A in susceptibility to syphilis was investigated. 221 healthy people with no history of syphilis and 137 people with syphilis cases were tested. RS5743708 causes a change from arginine to glutamine substitution at residue 753 (Arg753Gln) resulting in macrophage and bacterial peptides decreased response hence, leading to a host immune response that is attenuated. In syphilis patients, the Arg753Gln change had a much lower frequency than in controls. The findings implied that Arg753Gln (*TLR2*) does protect against syphilis development (16).

In 35 syphilis patients and 24 healthy volunteers, polymorphisms in the *IL10* gene (G>A) polymorphism at position −1084 and C>A polymorphism at position −592 were evaluated. Neurosyphilis was present in 31% of all syphilis cases. Patients with neurosyphilis had significantly greater levels of IL-10 than those with syphilis but not neurosyphilis. Higher cerebrospinal fluid *IL-10* levels were substantially correlated with the genotypes −1082 GG and −592 CC. Additionally, compared to those without neurosyphilis, people with neurosyphilis were found to have more of these genotypes (41). Furthermore, the researchers also investigated whether *IL17*A rs2275913 and rs3819024 were linked to *IL17A* mRNA expression and secretion in syphilis patients. Therefore, it was confirmed that the *IL17A* polymorphisms rs2275913 and rs3819024, as well as the haplotype including these two SNPs, influenced syphilis susceptibility (15). Not much research has been conducted on the association of host genes with syphilis and this calls for more investigations.

### Host genetic factors associated with *Trichomonas vaginalis* infections

*Trichomonas vaginalis* (TV) is a protozoan parasite that primarily causes trichomoniasis, the most prevalent non-viral STI. Trichomoniasis causes direct damage to the epithelium. The damage leads to micro-ulcerations, specifically in the vagina, cervix, urethra, and paraurethral glands. Globally, more than 100 million cases of trichomoniasis have been reported in men and women aged 15–49 years. The indirect association of TV infections with host genetic factors increases. Mostly, symptoms are not as bad and as a result, patients tend not to treat the infection leading to cervicitis and other long-term diseases. Cervical inflammation is one of the most common health problems among women worldwide, and it is caused by a variety of pathogens including CT, NG, TV, and HPV. TLR4 and TLR9 are known to play a key role in the induction of an inflammatory response to these pathogens. In a study performed on Indian women with cervicitis, TV infection was found at a higher rate (30.7%), than CT (1.5%), NG (2.3%), and HPV (4.6%). The rs11536889 CC (*TLR4*) and rs187084 TC (*TLR9*) genotypes had a larger distribution in cervicitis patients than in controls. Furthermore, as compared to controls, the rs11536889 C (*TLR4*) allele was found to increase the risk of cervicitis. TLR4 haplotype GCA and TLR9 haplotype GTA were associated with a lower and higher risk of cervicitis (17). The TLR4 polymorphisms, rs4986790 and rs4986791 are known to induce hyperresponsiveness to liposaccharides of gram-negative bacteria. Both polymorphisms are missense variants with rs4986790 G resulting in a change from aspartate to glycine and rs4986791 T resulting in a change from threonine to isoleucine. These SNPs were not shown to associate with TV in Iraqi women. It is worth noting that the wild type alleles for both SNPs are found at low frequency among Iraqi women (18). However, rs4986790 appeared to be associated with TV serostatus and risk for prostate cancer. Individuals with mutant G allele are likely to mount a weaker response to bacteria. A persistent TV infection may occur which could ascend to prostate cancer (42)). Not much has been published on Trichomonas vaginalis, this calls for more investigations to be conducted.

## Conclusion and future perspectives

Recognition of PAMPs through TLRs is essential for mounting immune response against STIs. The most significant associations were on TLRs which are the first line of defence of the immune response. STIs cause inflammatory responses by interacting with immune cells and the epithelial barrier at the infection site. Variation is genes associated with signalling this inflammatory response, which can alter the immune response against pathogens affecting an individual's susceptibility and the severity of the disease. Thus, it is important to understand the variation in immune response. Moreover, most studies on sexually transmitted infections have looked at the prevalence rather than the host genetic factors associated with these STIs. Thus, this review discussed current studies that have investigated the role of genetic variation in stimulating inflammatory response in prevalent STIs. The most prevalent bacterial (CT, NG and TP) and -protozoan (TV) infections were discussed. Only a few studies have investigated the role of host genetic variation associated with STI susceptibility and clinical outcomes. Better insights into the TLRs genetic variation, expression and association with the signalling pathways will help to gain additional knowledge of STIs-related immune therapy. Therefore, there is a need for more *in vivo*, *ex vivo* animal and site-specific studies to understand further the pathogenesis of infections and specific TLRs during various STIs. Furthermore, understanding variance within the TLR signalling pathway can also be used to develop biomarkers to predict an individual's response to an STI. Of the studies reviewed, most were conducted on African-American and Caucasian women. Non-Caucasian women are more likely to contract an STI. Understanding the genetic aspect as to why one ethnic group is more at risk compared to another, will minimize this disparity. Furthermore, African Americans are predominantly of Niger-Kordofanian ancestry. The studies conducted using African-American participants can not be extrapolated to all individuals of African ancestry as Africa has the highest level of genetic variation. Creating ethnic-specific biomarkers and therapy may curb the ethnic disparity observed in the prevalence of STIs. Furthermore, while females may be more vulnerable to STIs, only one study investigated host genetic variation associated with STIs in men. Females and Males have a slightly different immune response. Understanding the genetics associated with STIs in both Males and Females is essential for reducing the overall burden of STIs.

## References

[1] Taylor M Alonso-González M Gómez B Korenromp E Broutet N. World health organization global health sector strategy on sexually transmitted infections: an evidence-toaction summary for Colombia. Rev Colomb Obstet Ginecol. (2017) 68(3):193–201. 10.18597/RCOG.3071 31543554 PMC6754340

[2] WHO. Sexually transmitted infections (STIs) (2022). Available at: https://www.Who.Int/News-Room/Fact-Sheets/Detail/Sexually-Transmitted-Infections-(Stis)

[3] James C Harfouche M Welton NJ Turner KME Abu-Raddad LJ Gottlieb SL Herpes simplex virus: global infection prevalence and incidence estimates, 2016. Bull W H O. (2020) 98(5):315–29. 10.2471/BLT.19.237149 32514197 PMC7265941

[4] Deal C Cates W Peeling R Wald A. Long-term clinical sequelae of sexually transmitted infections in women. Emerg Infect Dis. (2004) 10(11):e2.

[5] Mwatelah R McKinnon LR Baxter C Abdool Karim Q Abdool Karim SS. Mechanisms of sexually transmitted infection-induced inflammation in women: implications for HIV risk. J Int AIDS Soc. (2019) 22(Suppl 6):e25346. 10.1002/JIA2.25346 31468677 PMC6715949

[6] Kawasaki T Kawai T. Toll-like receptor signaling pathways. Front Immunol. (2014) 5:461. 10.3389/fimmu.2014.00461 25309543 PMC4174766

[7] Yadav S Verma V Singh Dhanda R Yadav M. Insights into the toll-like receptors in sexually transmitted infections. Scand J Immunol. (2021) 93(1):e12954. 10.1111/sji.12954 32762084

[8] Youn H Hong K Yoo JW Lee CH. ICAM-1 expression in vaginal cells as a potential biomarker for inflammatory response. Biomarkers. (2008) 13(3):257–69. 10.1080/13547500701843338 18415799

[9] Abdelsamed H Peters J Byrne GI. Genetic variation in Chlamydia trachomatis and their hosts: impact on disease severity and tissue tropism. Future Microbiol. (2013) 8(9):1129–46. 10.2217/FMB.13.80 24020741 PMC4009991

[10] Taylor BD Darville T Ferrell RE Kammerer CM Ness RB Haggerty CL. Variants in toll-like receptor 1 and 4 genes are associated with chlamydia trachomatis among women with pelvic inflammatory disease. J Infect Dis. (2012) 205(4):603–9. 10.1093/infdis/jir822 22238472 PMC3266128

[11] Verweij SP Karimi O Pleijster J Lyons JM De Vries HJC Land JA TLR2, TLR4 and TLR9 genotypes and haplotypes in the susceptibility to and clinical course of Chlamydia trachomatis infections in Dutch women. Pathog Dis. (2018) 74(1):1–8. 10.1093/femspd/ftv107 PMC4882084 26568059

[12] Brankoví I Van Ess EF Noz MP Wiericx WA Spaargaren J Morré SA. NOD1 in contrast to NOD2 functional polymorphism influence Chlamydia trachomatis infection and the risk of tubal factor infertility. FEMS Pathog Dis. (2015) 73(1):1–9. 10.1093/femspd/ftu028 PMC4542905 25854006

[13] Jukema JB Hoenderboom BM van Benthem BH Van der Sande MA de Vries HJ Hoebe CJ Can previous associations of single nucleotide polymorphisms in the tlr2, nod1, cxcr5, and il10 genes in the susceptibility to and severity of chlamydia trachomatis infections be confirmed? Pathogens. (2021) 10(1):48. 33430411 10.3390/pathogens10010048 PMC7827792

[14] Taylor BD Darville T Ferrell RE Kammerer CM Ness RB Haggerty CL. Racial variation in toll-like receptor variants among women with pelvic inflammatory disease. J Infect Dis. (2012) 207(6):940–6. 10.1093/infdis/jis922 23255565 PMC3571443

[15] Hu WL Ren H Xu BF Zhang JP Zhang RL Wang QQ Evaluation of IL-17A, IL-17F, IL-23R, VDR, CCL2, CCL5, CCR2, and CCR5 gene polymorphisms and expression in Chinese individuals with syphilis. J Cell Biochem. (2018) 119(12):10151–64. 10.1002/jcb.27352 30171709

[16] Grillová L Musilová J Janečková K Pospíšilová P Kuklová I Woznicová V The Arg753Gln polymorphism of toll-like receptor 2 has a lower occurrence in patients with syphilis, suggesting its protective effect in Czech and Slovak individuals. Infect Immun. (2021) 89(1):e00503–20. 10.1128/IAI.00503-20/SUPPL_FILE/IAI.00503-20-S0001.PDF PMC7927923 33077622

[17] Chauhan A Pandey N Desai A Raithatha N Patel P Choxi Y Association of TLR4 and TLR9 gene polymorphisms and haplotypes with cervicitis susceptibility. PLoS ONE. (2019) 14(7):1–15. 10.1371/journal.pone.0220330 PMC6668796 31365550

[18] Chaloob F Abdul-Mohsen AS. Association of toll-like receptor 4 gene oolymorphism with Trichomonas vaginalis infection in Iraqi women. Med J Babylon. (2014) 11(1):84–91.

[19] Huai P Li F Chu T Liu D Liu J Zhang F. Prevalence of genital Chlamydia trachomatis infection in the general population: a meta-analysis. BMC Infect Dis. (2020) 20(1):1–8. 10.1186/s12879-020-05307-w PMC7414538 32770958

[20] Geisler WM Chow JM Schachter J Mccormack WM. Pelvic examination findings and Chlamydia trachomatis infection in asymptomatic young women screened with a nucleic acid amplification test. Sex Transm Dis. (2007) 34(6):335–8. 10.1097/01.OLQ.0000240344.20665.63 17028510

[21] Barnes AB Keener RM Schott BH Wang L Valdivia RH Ko DC. Human genetic diversity regulating the TLR10/TLR1/TLR6 locus confers increased cytokines in response to Chlamydia trachomatis. Hum Genet Genomics Adv. (2022) 3(1):100071. 10.1016/j.xhgg.2021.100071 PMC8756536 35047856

[22] Malogajski J Branković I Land JA Thomas PPM Morré SA Ambrosino E. The potential role for host genetic profiling in screening for chlamydia-associated tubal factor infertility (TFI)—new perspectives. Genes (Basel). (2019) 10(6):410. 10.3390/genes10060410 31142036 PMC6627277

[23] Iwasaki A Medzhitov R. Toll-like receptor control of the adaptive immune responses. Nat Immunol. (2004) 5(10):987–95. 10.1038/ni1112 15454922

[24] Syed Sameer A & Nissar S. *Toll-like receptors (TLRs): structure, functions*, signaling, and role of their polymorphisms in colorectal cancer susceptibility. Biomed Res Int. (2021) 2021. 10.1155/2021/1157023 PMC8452412 34552981

[25] Zhang Y Bliska JB. Role of toll-like receptor signaling in the apoptotic response of macrophages to Yersinia infection. Infect Immun. (2003) 71(3):1513–9. 10.1128/IAI.71.3.1513-1519.2003 12595470 PMC148878

[26] Karimi O Ouburg S De Vries HJC Peña AS Pleijster J Land JA TLR2 haplotypes in the susceptibility to and severity of Chlamydia trachomatis infections in Dutch women. Drugs Today. (2009) 45(Suppl B):67–74. 20011697

[27] Yadav S Verma V Singh Dhanda R Yadav M. Insights into the toll-like receptors in sexually transmitted infections. Scand J Immunol. (2021) 93(1):1–17. 10.1111/sji.12954 32762084

[28] Darville T O’Neill JM Andrews CW Nagarajan UM Stahl L Ojcius DM. Toll-like receptor-2, but not toll-like receptor-4, is essential for development of oviduct pathology in chlamydial genital tract infection. J Immunol. (2003) 171(11):6187–97. 10.4049/jimmunol.171.11.6187 14634135

[29] Stevens JS Criss AK. Pathogenesis of Neisseria gonorrhoeae in the female reproductive tract: neutrophilic host response, sustained infection, and clinical sequelae. Curr Opin Hematol. (2018) 25(1):13–21. 10.1097/MOH.0000000000000394 29016383 PMC5753798

[30] El-Zayat SR Sibaii H Mannaa FA. Toll-like receptors activation, signaling, and targeting: an overview. Bull Natl Res Cent. (2019) 43:187. 10.1186/s42269-019-0227-2

[31] Moreira LO Zamboni DS. NOD1 and NOD2 signaling in infection and inflammation. Front Immunol. (2012) 3:328. 10.3389/fimmu.2012.00328 23162548 PMC3492658

[32] Welter-Stahl L Ojcius DM Viala J Girardin S Liu W Delarbre C Stimulation of the cytosolic receptor for peptidoglycan, Nod1, by infection with Chlamydia trachomatis or Chlamydia muridarum. Cell Microbiol. (2006) 8(6):1047–57. 10.1111/J.1462-5822.2006.00686.X 16681844

[33] Iyer SS Cheng G. Role of interleukin 10 transcriptional regulation in inflammation and autoimmune disease. Crit Rev Immunol. (2012) 32(1):23–63. 10.1615/critrevimmunol.v32.i1.30 22428854 PMC3410706

[34] Vermund SH Geller AB Crowley JS. Sexually transmitted infections. Sex Transm Infect. (2022):1–750. 10.17226/25955

[35] Jarvis GA Li J Swanson KV. Invasion of human mucosal epithelial cells by Neisseria gonorrhoeae upregulates expression of intercellular adhesion molecule 1 (ICAM-1)†. Infect Immun. (1999) 67(3):1149–56. 10.1128/IAI.67.3.1149-1156.1999 10024555 PMC96441

[36] Mikamo H Yamagishi Y Sugiyama H Sadakata H Miyazaki S Sano T High glucose-mediated overexpression of ICAM-1 in human vaginal epithelial cells increases adhesion of Candida albicans. J Obstet Gynaecol. (2018) 38(2):226–30. 10.1080/01443615.2017.1343810 28920516

[37] Geisler WM Wang C Tang J Wilson CM Crowley-Nowick PA Kaslow RA. Immunogenetic correlates of neisseria gonorrhoeae infection in adolescents. Sex Transm Dis. (2008) 35:656–61. 10.1097/OLQ.0b013e31816b6593 18496418 PMC2705886

[38] Satyaputra F Hendry S Braddick M Sivabalan P Norton R. The laboratory diagnosis of syphilis. J Clin Microbiol. (2021) 59(10):e0010021. 10.1128/JCM.00100-21 33980644 PMC8451404

[39] Marra CM Sahi SK Tantalo LC Ho EL Dunaway SB Jones T Toll-like receptor polymorphisms are associated with increased neurosyphilis risk. Sex Transm Dis. (2014) 41(7):440–6. 10.1097/OLQ.0000000000000149 24922103 PMC4414322

[40] Hubner RA Houlston RS. Single nucleotide polymorphisms and cancer susceptibility. Mol Basis Hum Cancer. (2016) 8(66):231–9. 10.1007/978-1-59745-458-2_14

[41] Pastuszczak M Jakiela B Jaworek AK Wypasek E Zeman J Wojas-Pelc A. Association of interleukin-10 promoter polymorphisms with neurosyphilis. Hum Immunol. (2015) 76(7):469–72. 10.1016/J.HUMIMM.2015.06.010 26100683

[42] Chen YC Huang YL Platz EA Alderete JF Zheng L Rider JR Prospective study of effect modification by toll-like receptor 4 variation on the association between trichomonas vaginalis serostatus and prostate cancer. Cancer Causes Control. (2013) 24:175–80. 10.1007/s10552-012-0103-y 23179660 PMC3549464

